# ALMT12: A dual regulator of S-type anion transport in guard cells?

**DOI:** 10.1093/plphys/kiaf635

**Published:** 2025-12-07

**Authors:** Bo Xu

**Affiliations:** Assistant Features Editor, Plant Physiology, American Society of Plant Biologists; ARC Centre of Excellence in Plants for Space, and School of Agriculture, Food and Wine, Waite Research Institute, University of Adelaide, Glen Osmond, SA 5064, Australia

Stomata are microscopic pores on the leaf surface surrounded by pairs of guard cells, controlling plant gaseous exchange between leaf inner airspace and atmosphere. Through these pores, plants absorb carbon dioxide (CO_2_) for photosynthesis and release oxygen (O_2_) and water vapor (H_2_O) via transpiration (Blatt 2024). To cope with environmental changes, plants fine-tune transmembrane ion flux across diverse ion channels within guard cells. This modulation adjusts guard-cell turgor pressure and stomatal pore sizes to optimize CO_2_ assimilation against water loss, a critical balance for plant growth (Blatt 2024). For instance, elevated CO_2_ promotes stomatal closure (Jalakas et al. 2021; Blatt 2024). In this process, the stomatal closure is modulated by activation of plasma membrane–localized slow (S)- and rapid (R)-type anion channels, respectively encoded by SLOW ANION CHANNEL 1 (SLAC1) and ALUMINUM-ACTIVATED MALATE TRANSPORTER 12/QUICK-ACTIVATING ANION CHANNEL 1 (ALMT12/QUAC1) (Jalakas et al. 2021). *Arabidopsis thaliana* mutants lacking either *SLAC1* or *ALMT12/QUAC1* impair stomatal closure in response to high CO_2_ (800 ppm) compared with wild-type (WT) plants. Notably, the *slac1/almt12* double mutant almost abolished CO_2_-induced stomatal response completely, highlighting their critical roles in mediating CO_2_-responsive anion efflux from guard cells (Jalakas et al. 2021). Among these two, SLAC1 plays a more prominent role than ALMT12/QUAC1, as *slac1* displays a more severe closure defect than *almt12* under high CO_2_ conditions (Jalakas et al. 2021).

Guard-cell ion channels can also function through direct physical interactions between proteins localized within the membrane (Liu et al. 2023). For example, SLAC1 HOMOLOG 3 (SLAH3)—another S-type anion channel not involved in CO_2_ responses (Jalakas et al. 2021)—has been shown to physically interact with GUARD-CELL OUTWARD RECTIFYING K^+^ CHANNEL (GORK). This interaction partially inhibits GORK-mediated potassium (K^+^) transport, while SLAH3 retains its nitrate (NO_3_^−^) transport activity in *Xenopus laevis* oocytes (Liu et al. 2023). Beyond guard cells, *SLAH3* and *GORK* are also expressed in roots, where they operate collaboratively to modulate K^+^ and NO_3_^−^ homeostasis (Liu et al. 2023). Additionally, computational modeling with OnGuard platform (Minguet-Parramona et al. 2016) has shown that R-type currents depend on the S-type currents to bias the membrane before triggering action potential–like oscillations through the R-type currents. These oscillations are important for accelerating closure. However, it remains unknown whether S-type and R-type anion channels, such as SLAC1 and ALMT12/QUAC1, also function in guard cells through physical interactions.

In a recent study published in *Plant Physiology*, Lin et al. (2025) reported that ALMT12/QUAC1 inhibits OPEN STOMATA 1 (OST1)-activated SLAC1-mediated anion efflux in *X. laevis* oocytes. Protein-protein interaction assays across multiple systems suggested a direct physical interaction between SLAC1 and ALMT12/QUAC1, supporting the idea that inhibition occurs through direct binding. Interestingly, 2 site-direct SLAC1 mutants—SLAC1^F450A^ and SLAC1^T513D^, which conduct S-type anion transport without OST1 phosphorylation—were also inhibited by ALMT12/QUAC1. This indicates that SLAC1 inhibition by ALMT12/QUAC1 does not require OST1 (Lin et al. 2025).

Whole-cell patch-clamp assays extended these findings to native Arabidopsis guard cells. Under basal conditions, S-type anion currents were similar between WT and *almt12* mutants. However, when activated by abscisic acid (ABA), calcium (Ca^2+^), or bicarbonate (HCO_3_^−^, as part of CO_2_ treatment), *almt12* guard cells displayed significantly greater S-type currents (Lin et al. 2025). Given the critical roles of SLAC1-mediated transport in stomatal closure (Jalakas et al. 2021), this implied that loss of *ALMT12/QUAC1* might accelerate stomatal closure. Yet, epidermal peel assays showed the opposite: ABA- and Ca^2+^-induced stomatal closure was actually reduced in *almt12* compared with WT (Lin et al. 2025) although consistent with previous research (Minguet-Parramona et al. 2016; Jalakas et al. 2021). In contrast, CO_2_-induced closure was enhanced in *almt12*, aligning with the patch-clamp results (Lin et al. 2025). On this basis, Lin et al. (2025) proposed that ALMT12/QUAC1 acts as a positive regulator of stomatal responses to environmental CO_2_ for balancing carbon fixation, with *almt12* plants exhibiting reduced biomass compared with WT.

One puzzling observation is that *almt12* displayed enhanced stomatal closure on epidermal peels under 10 mM HCO_3_^−^ treatment, contrasting earlier reports that *almt12* attenuates stomatal closure at high (800 ppm) CO_2_ (Meyer et al. 2010; Jalakas et al. 2021; Lin et al. 2025). The discrepancy between epidermal peel assays and intact plants suggests an additional layer of regulation. Another discovery in Arabidopsis may offer a complementary observation. Xu et al. (2021) identified GABA as a stomatal guard-cell signaling molecule that inhibits light-induced stomatal opening and dark-induced stomatal closure via negatively regulation of ALMT9 and ALMT12/QUAC1, respectively. Notably, the GABA-suppressed stomatal closure via inhibiting ALMT12/QUAC1 was observed on epidermal peels but disappeared in intact leaves, likely because of mesophyll cues missing (Xu et al. 2021). Since mesophyll cells play a key role in stomatal CO_2_ sensing (Mott et al. 2008; Fujita et al. 2013), the absence of mesophyll regulation in patch-clamp and stomatal aperture assays may explain why *almt12* CO_2_ responses differ on epidermal peels versus in intact plants (Meyer et al. 2010; Lin et al. 2025). Together, these observations suggest that the role of ALMT12/QUAC1 in stomatal regulation probably depends on additional mesophyll-derived signals that remain unknown. Another puzzling observation is that while the N-terminal half of ALMT12 failed to interact with SLAC1, it nonetheless fully abolished SLAC1-mediated anion currents. This apparent contradiction between interaction and inhibition warrants further investigation.

The study by Lin et al. (2025) adds another complex layer of interplay between R- and S-type anion transport in guard cells. ALMT12/QUAC1 mediates (R-type) malate efflux from guard cells (Meyer et al. 2010), and apoplastic malate can activate SLAC1-mediated S-type anion transport, including SLAC1^F450A^ and SLAC1^T513D^ mutants (Mimata et al. 2022). This raises the intriguing possibility that ALMT12/QUAC1 may regulate SLAC1 in 2 ways: direct S-type transport inhibition through SLAC1-ALMT12/QUAC1 interaction and indirect S-type transport activation by ALMT12/QUAC1-modulated extracellular malate pools that feedback on SLAC1 regulation in guard cells (Fig.). Such dual structural and metabolic regulatory roles highlight ALMT12/QUAC1 as a central integrator of guard-cell signaling, warranting future work to unravel how it fine-tunes stomatal behavior.

**Figure.**
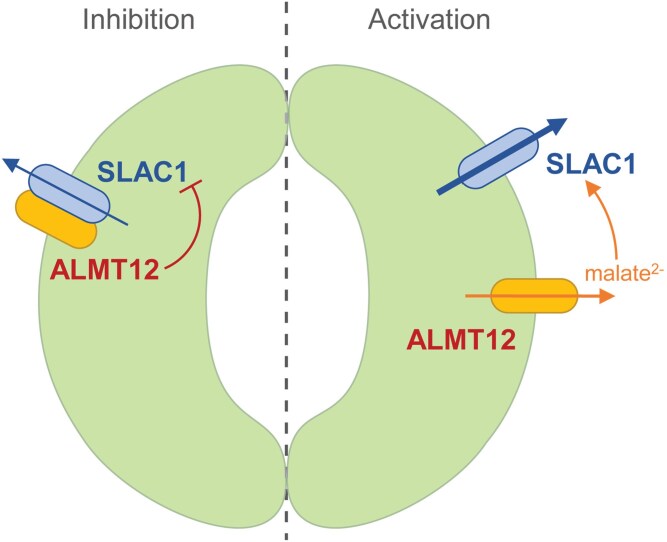
Proposed model of ALMT12-modulated inhibition and activation of anion transport through SLAC1 in guard cells. ALMT12/QUAC1 directly interacts with SLAC1 to suppress SLAC1-mediated S-type anion efflux in guard cells (inhibition) (Lin et al. 2025). In contrast, ALMT12/QUAC1-catalyzed malate^2−^ efflux increases extracellular malate accumulation, which subsequently activates SLAC1 (activation) (Meyer et al. 2010; Mimata et al. 2022).

## Data Availability

No new data were generated or analyzed in support of this research.
